# Crystal structures of the *Bacillus subtilis* prophage lytic cassette proteins XepA and YomS

**DOI:** 10.1107/S2059798319013330

**Published:** 2019-11-01

**Authors:** Stefanie Freitag-Pohl, Andrius Jasilionis, Maria Håkansson, L. Anders Svensson, Rebeka Kovačič, Martin Welin, Hildegard Watzlawick, Lei Wang, Josef Altenbuchner, Magdalena Płotka, Anna Karina Kaczorowska, Tadeusz Kaczorowski, Eva Nordberg Karlsson, Salam Al-Karadaghi, Björn Walse, Arnthór Aevarsson, Ehmke Pohl

**Affiliations:** a Department of Chemistry, Durham University, South Road, Durham DH1 3LE, England; b Division of Biotechnology, Lund University, PO Box 124, SE-221 00 Lund, Sweden; c SARomics Biostructures, Scheelevägen 2, SE-223 63 Lund, Sweden; d Institut for Industrial Genetics, University of Stuttgart, Allmandring 31, 70569 Stuttgart, Germany; e Department of Microbiology, Faculty of Biology, University of Gdańsk, Kladki 24, 80-824 Gdańsk, Poland; f Collection of Plasmids and Microorganisms, Faculty of Biology, University of Gdańsk, Kladki 24, 80-824 Gdańsk, Poland; g Matis, Vinlandsleid 12, 113 Reykjavik, Iceland; h Department of Biosciences, Durham University, South Road, Durham DH1 3LE, England

**Keywords:** prophage, Virus-X Consortium, lytic enzymes, lytic cassette proteins, *Bacillus subtilis*, XepA, YomS

## Abstract

As part of the Virus-X Consortium that aims to identify and characterize novel proteins and enzymes from bacteriophages and archaeal viruses, the genes of the putative lytic proteins XepA from *Bacillus subtilis* prophage PBSX and YomS from prophage SPβ were cloned and the proteins were subsequently produced and functionally characterized. In order to elucidate the role and the molecular mechanism of XepA and YomS, the crystal structures of these proteins were solved at resolutions of 1.9 and 1.3 Å, respectively. XepA consists of two antiparallel β-sandwich domains connected by a 30-amino-acid linker region. A pentamer of this protein adopts a unique dumbbell-shaped architecture consisting of two discs and a central tunnel. YomS (12.9 kDa per monomer), which is less than half the size of XepA (30.3 kDa), shows homology to the C-terminal part of XepA and exhibits a similar pentameric disc arrangement. Each β-sandwich entity resembles the fold of typical cytoplasmic membrane-binding C2 domains. Only XepA exhibits distinct cytotoxic activity *in vivo*, suggesting that the N-terminal pentameric domain is essential for this biological activity. The biological and structural data presented here suggest that XepA disrupts the proton motive force of the cytoplasmatic membrane, thus supporting cell lysis.

## Abstract 2

The lytic cassette proteins XepA and YomS from *Bacillus subtilis* prophages have been characterized and it was found that only XepA establishes cytotoxic activity in plaque assays. The crystal structures of both proteins show a unique pentameric assembly, in which YomS adopts a very similar fold to the C-terminal domain of the XepA dumbbell pentamer. The overall architecture of XepA, with the N-terminal domain subunits resembling cytoplasmic membrane-binding C2-domain folds, suggests that any lytic functionality could be based on disruption of the proton motive force of the cytoplasmic membrane, which induces cell lysis.

## 1. Introduction   

Bacteriophages are viruses that infect bacteria and replicate within their host cells. Prophage DNA remains integrated in the bacterial DNA until the proliferation of new phages is triggered, and ultimately the host cell is lysed to release the phage progeny. Bacteriophages employ a versatile and long-evolved proteome which is an invaluable source of proteins with significant potential biotechnological applications. For example, depolymerases have been explored for antiviral strategies (Hsieh *et al.*, 2017 ▸), whilst tail fibre proteins have been investigated for biosensor development (Denyes *et al.*, 2017 ▸) and as cofactors to increase the specificity of PCR-based DNA amplification (Stefanska *et al.*, 2014 ▸). Phage proteins possess enormous potential as bacterial markers, drug transporters and for vaccine development (Drulis-Kawa *et al.*, 2015 ▸; Plotka *et al.*, 2015 ▸). In addition, the global threat of antibiotic resistance emphasizes the requirement for more novel tools and understanding in order to counteract bacterial infection. Lytic enzymes are increasingly being recognized as highly effective antibacterials (Briers, 2019 ▸). As the multinational Virus-X Consortium (http://virus-x.eu/) has been established to explore the function and structure of novel phage enzymes with biotechnological and biomedical potential, we chose two potentially lytic enzymes from two *Bacillus subtilis* 168 defective prophages as a starting point for investigations: XepA (PBSX exported protein, also known as XkdY or P31) from the PBSX prophage and YomS from the SPβ prophage (Longchamp *et al.*, 1994 ▸).

**Figure 1:**
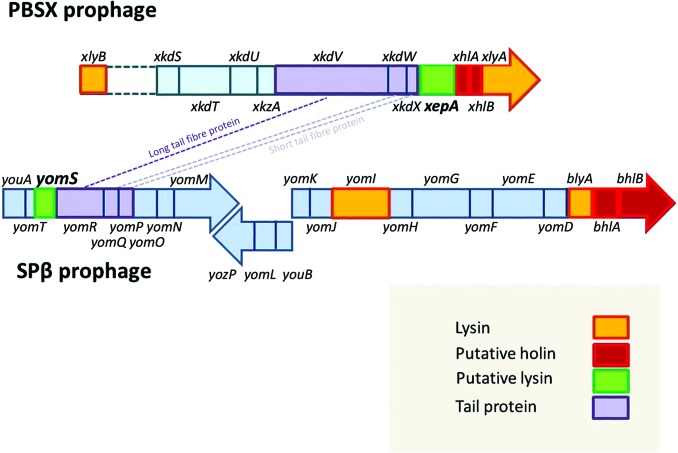
Gene organization of the PBSX and SPβ prophage late operons in the area of the lytic systems (red) described. Whereas *xepA* in prophage PBSX is located in the direct vicinity of the lytic entity (*xhlA*, *xhlB*, *xlyA*), *yomS* in SPβ is further removed from the *blyA*, *bhlA*, *bhlB* region. The cassettes containing *xkdV*, *xkdW*, *xkdX* and *yomR*, *yomQ*, *yomP* encode structural tail proteins (purple). The *xlyB* gene is located further upstream in the PBSX genome.

Prophage generation can be induced in *B. subtilis* by the addition of mitomycin C or thermally. XepA appears 20 min after induction and its concentration increases steadily until cell lysis. It is not present in the mature phage particles, but is exported beyond the cytoplasmic membrane during phage development, suggesting that the protein is involved in cell-wall metabolism or degradation (Mauël & Karamata, 1984 ▸). The genome of both phages, PBSX and SPβ, and their cell-lysis systems (Fig. 1 ▸) have been described previously (Wood *et al.*, 1990 ▸; Lazarevic *et al.*, 1999 ▸). Phage-induced lysis was reported to rely on the holin–lysin dyad, in which holins make the cytoplasmic membrane (CM) permeable and a lysin attacks the host peptidoglycan (PG). All phage lysins can together mostly be described as peptidoglycan hydrolases. However, they have a high diversity and attack different bonds in the PG and thus represent peptidases, amidases and glucosidases, as well as transglycosylases (Smith *et al.*, 2000 ▸; Low *et al.*, 2011 ▸). Although XepA was described as being part of the host cell lysis system of PBSX together with the *N*-acetylmuramoyl-l-alanine amidase XlyA (also known as P32) and the two putative holins XhlA and XhlB (also known as XpaB), the biological role and molecular mechanism of XepA remain elusive (Longchamp *et al.*, 1994 ▸). The contributions of each member of the lytic system (XepA, XlyA, XhlA and XhlB) were further investigated by variation and deletion of their genes in the PBSX late operon and subsequent expression in *B. subtilis* (Krogh *et al.*, 1998 ▸). The results showed that the holin XhlA is essential for cell lysis, whereas the other three proteins alone cannot induce cell lysis. XlyA was shown to have a strong affinity for teichoic acid-containing cell walls and to play the major role in the degradation of the host cell wall (Mauël & Karamata, 1984 ▸). More recently, XlyA was also reported to be essential in membrane-vesicle formation in *B. subtilis* (Toyofuku *et al.*, 2017 ▸). Another gene of a putative member of the PBSX lytic system lies upstream, *xlyB* (also known as *yjpB*), which encodes a putative amidase (Smith *et al.*, 2000 ▸).

In the SPβ prophage the host cell lysis system consists of the *N*-acetylmuramoyl-l-alanine amidase BlyA (also known as YomC), BhlA and BhlB as putative holins and YomI (also known as CwlP), which has been described as a virion-associated peptidoglycan hydrolase (Sudiarta *et al.*, 2010 ▸; Rodríguez-Rubio *et al.*, 2013 ▸). YomS is further removed from the genes of the lytic system in the SPβ prophage operon and is located next to the structural tail protein-encoding cassette (Fig. 1 ▸). To date, the only structural detail described for the lytic machinery of both prophages is the X-ray structure of the catalytic domain of XlyA (Low *et al.*, 2011 ▸). In order to elucidate their role in the lytic cassette of *B. subtilis* prophages, we cloned and produced XepA from the PBSX prophage and YomS from the SPβ prophage. To shed light on the function of XepA and YomS, we concentrated our efforts on gaining high-resolution structural information on the putative lysins, which we now present here together with initial functional studies.

## 2. Materials and methods   

### 2.1. Cloning, protein production, purification and initial characterization   

In addition to XepA and YomS, the lysins XlyA and XlyB were cloned and produced for inclusion in lytic assays. To express the respective genes in *Escherichia coli*, the coding regions for the *xepA*, *xlyA*, *xlyB* and *yomS* genes were amplified by PCR with genomic DNA from *B. subtilis* 168 serving as a template. The gene-specific primers S11669 (gcggatccGTGAAGTATCAATATGAATTTCCTC) and S11670 (gcctgtacaTTATGAAACCGCGGTCCCTTTTAC) for *xepA*; S11667 (gcggatccGTTAACATTATTCAAGACTTTATTC) and S11668 (gcctgtacaTCAGCTTAATTGCGCTGCGAT) for *xlyA*; S11665 (gcggatccAGCATTCCAGTAAAGAAAAATTTG) and S11666 (gcctgtacaTTACAGCTTTTCCTCCATCTTC) for *xlyB*; and S11675 (gcggatccACAGAAACGACTGAAAATGTCG) and S11676 (gcctgtacaTTAACTCACCACAATCCCTTTAAC) for *yomS* were used in PCR reactions, which introduced a BamHI site into the N-terminal gene sequence and a BsrGI restriction site just behind the stop codon. The respective PCR fragments were cloned in-frame into the rhamnose-inducible vector pJOE5751 (Wegerer *et al.*, 2008 ▸) and resulted in the expression plasmids pHWG1186 for His_6_-*xepA*, pHWG1185 for His_6_-*xlyA*, pHWG1184 for His_6_-*xlyB* and pHWG1189 for His_6_-*yomS*.

Deletion mutants of *xepA* were constructed by PCR amplification using pHWG1186 as a template. The N-terminal *xepA* domain without the central tunnel region was amplified with the primers S11669 (see above) and S12646 (gcctgtacaTTAACGCACATCCATCTCACCCG) and yielded a 441 bp fragment. The N-terminal *xepA* domain with the tunnel region was amplified with the primers S11669 (see above) and S12647 (gcctgtacaTTAAGCCTCGACTTTCAGCCGTC), yielding a 520 bp fragment, while for amplification of the C-domain of *xepA* the primers S12648 (gcggatccGTCAATGAAAAAACGCCTTTACA) and S11670 (see above) were used and resulted in a 325 bp fragment. The respective PCR fragments, which contained additional BamHI and BsrGI restriction sites, respectively, were cloned in-frame into pJOE5751 as mentioned above and resulted in pHWG1319 for His_6_-*xepA* without the tunnel region (XepA_N), pHWG1320 for His_6_-*xepA* with the tunnel region (XepA_NL) and pHWG1321 for His_6_-*xepA*-C-domain (XepA_C). After transformation of the plasmids in *E. coli* JM109 cells, 15 ml precultures were grown overnight in LB–ampicillin at 37°C. After inoculating a 1 l LB–ampicillin culture with the preculture, the bacteria were grown for 4 h to an OD_600 nm_ of 0.6–0.8. Protein production was induced by the addition of rhamnose to a final concentration of 0.2% at 30°C. The culture was incubated overnight and was subsequently centrifuged at 1300*g* at 4°C. The pellet was resuspended in lysis buffer (20 m*M* Tris–HCl pH 7.5) with added protease inhibitor. After sonication (2–15 min, 40%, 4°C) the supernatant was filtered and was subsequently loaded onto a HisTrap HP affinity column, which was subjected to an imidazole-gradient FPLC separation. The protein was dialyzed into 10 m*M* ammonium bicarbonate buffer pH 7.5 and the molecular weight was verified by ESI-MS and SDS–PAGE. For YomS, selenomethionine (SeMet) needed to be incorporated in the protein sequence for crystallographic structure solution. Accordingly, the YomS expression construct was transformed into the methionine-auxotrophic *E. coli* strain B834(DE3). SeMet incorporation was performed following the general recommendations (Walden, 2010 ▸). YomS was heterogeneously overexpressed by cultivating the expression strain in mAT/ampicillin medium (de Maré *et al.*, 2005 ▸) supplemented with SeMet to a final concentration of 50 mg l^−1^. Harvesting, purification and analysis of the SeMet-YomS variant was performed as described for the native proteins.

### 2.2. Thermal shift assay   

Thermal shift assays were performed to identify stabilizing conditions for protein storage and crystallization (Niesen *et al.*, 2007 ▸) using the Durham Screens (Bruce *et al.*, 2019 ▸). Briefly, 1 ml protein solution at approximately 1 mg ml^−1^ in 10 m*M* Tris–HCl buffer pH 7.5 was mixed with 4 µl SYPRO Orange dye (5000× in DMSO) and 10 µl was pipetted into a 96-well PCR plate. 10 µl of the screens were added to the wells and the plate was sealed with thermostable film. The plate was centrifuged at 160*g* at 4°C for 2 min and was subsequently placed in a Real-Time PCR machine for melting-temperature experiments. Data from the thermal shift assay screen were analysed using in-house *Microsoft Excel* scripts and *NAMI* (Grøftehauge *et al.*, 2015 ▸). To determine an optimal buffer for the crystallization of XepA and YomS, additional thermal shift assays were performed with a reduced 20-condition screen (Niesen *et al.*, 2007 ▸). In these screens, several buffers at 100 m*M* with different pH values were used and the sodium chloride and glycerol concentrations were varied. For the small buffer screen the XepA and YomS proteins were diluted to 0.13 mg ml^−1^ in 10 m*M* HEPES pH 7.5 and 6.7× SYPRO Orange. 8 µl of a 4× concentration of buffer screen was dispensed into a 96-well PCR plate and 24 µl of the protein/SYPRO Orange mixture was added to each well with the buffer screen. The temperature range for the thermal shift analysis was 25–95°C (1°C steps per minute) and the fluorescence was measured after each increment. In addition, thermal shift assays with all target proteins and a number of glycosides were performed using the same protocol to assess ligand binding.

### 2.3. Crystallization   

Initial crystallization experiments were performed using a range of commercially available crystallization screens. XepA crystallized in several crystal forms. Crystal form I (blocks, ∼0.1 × 0.1 × 0.1 mm) was obtained at 4°C using a protein solution consisting of 15 mg ml^−1^ XepA in 20 m*M* bis–Tris–HCl pH 6.5, 50 m*M* NaCl and was grown in a self-seeded drop with a reservoir consisting of 0.1 *M* sodium acetate pH 5.0, 6%(*w*/*v*) PEG 4000. Co-crystals with a terbium compound (crystal form II; ∼0.03 × 0.03 × 0.2 mm) were obtained by adding 100 µl protein solution (as above) to 0.6 mg of a Tb cluster compound (Crystallophore from Molecular Dimensions; Engilberge *et al.*, 2017 ▸) and setting up self-seeded crystallization experiments at 4°C with the reservoir consisting of 0.1 *M* sodium acetate pH 5.2, 7%(*w*/*v*) PEG 4000. Crystallization experiments were set up in sitting drops in MRC 3-well plates using a Mosquito robot (TTP Labtech). Long needles (crystal form II, ∼0.05 × 0.05 × 0.3 mm) were grown using 0.1 *M* magnesium acetate, 0.1 *M* potassium chloride, 12% PEG Smear High (Molecular Dimensions), 0.1 *M* MES pH 5.5 and 4 mg ml^−1^ XepA in Tris buffer and were crystallized manually in vapour-diffusion sitting drops. YomS crystals (∼0.03 × 0.2 × 0.3 mm) were obtained at 20°C using 13.4 mg ml^−1^ YomS in 20 m*M* MES pH 6.0, 150 m*M* sodium chloride mixed with an equal volume of 0.1 *M* cacodylate pH 5.5, 0.2 *M* ammonium nitrate, 18%(*w*/*v*) PEG Smear Low (Molecular Dimensions). Selenomethionine-containing YomS crystals (∼0.03 × 0.1 × 0.2 mm) were grown from 6 mg ml^−1^ SeMet-YomS under the same conditions by self-seeding techniques.

### 2.4. Data collection, structure solution and refinement   

All crystals were transferred into cryosolution and flash-cooled in liquid nitrogen before data collection (Garman, 2003 ▸). XepA form I crystals were transferred into 0.1 *M* sodium acetate pH 5.0, 9%(*w*/*v*) PEG 4000, 50 m*M* sodium chloride, 30%(*v*/*v*) glycerol. Tb-derivatized XepA form II crystals were soaked in a solution with 0.6 mg terbium cluster added to 10–100 µl cryosolution for approximately 1 min. Native YomS crystals were cryoprotected in 0.1 *M* sodium cacodylate pH 5.5, 0.2 *M* ammonium nitrate, 22%(*w*/*v*) PEG Smear Low, 25% glycerol, 30 m*M* sodium chloride. SeMet-YomS crystals were transferred into 0.1 *M* sodium cacodylate pH 5.5, 0.2 *M* ammonium nitrate, 20%(*w*/*v*) PEG Smear Low, 25% PEG 400. Data were collected using a PILATUS pixel-array detector (Broennimann *et al.*, 2006 ▸) on beamlines I03, I04 and I24 at the Diamond Light Source (DLS), Didcot, England (YomS and XepA), as well as on beamline P13 at EMBL/DESY (SeMet-YomS; Cianci *et al.*, 2017 ▸). All native data were processed using either *autoPROC* (Vonrhein *et al.*, 2011 ▸) and *STARANISO* or *XDS* (Kabsch, 2010 ▸) followed by *POINTLESS* and *AIMLESS* as implemented in *CCP*4 (Winn *et al.*, 2011 ▸) or the *xia*2 software pipeline (Winter *et al.*, 2013 ▸). The anomalous data were processed in *XDS* and scaled in *XSCALE*, and *AIMLESS* was used to produce an MTZ file that was fed into the *CRANK*2 pipeline (Pannu *et al.*, 2011 ▸), which uses *SHELXC*/*D*/*E* (Sheldrick, 2010 ▸) for automatic phasing by single-wavelength anomalous diffraction (SAD). For XepA, several data sets were collected at the terbium peak and the two most isomorphous data sets were scaled together to enhance the anomalous signal. The higher resolution native data set from crystal form I was subsequently used for structure refinement (Table 1 ▸). XepA crystal form I and the native crystal form II data were solved employing the SAD solution as a molecular-replacement model with *Phaser* (McCoy *et al.*, 2007 ▸). In the case of YomS, the structure was determined using a single data set collected at the selenium peak. All structural models were refined against the diffraction data with *REFMAC*5 (Murshudov *et al.*, 2011 ▸) using local noncrystallographic symmetry restraints when appropriate (Usón *et al.*, 1999 ▸) or with *BUSTER* (Smart *et al.*, 2012 ▸). All model building and evaluation was performed with *Coot* (Emsley *et al.*, 2010 ▸). The final models were checked using *MolProbity* (Chen *et al.*, 2015 ▸). Least-squares superpositions of C^α^ atoms were performed with *RAPIDO* (Mosca & Schneider, 2008 ▸) or *CCP*4*mg* (McNicholas *et al.*, 2011 ▸). Further crystallographic data are summarized in Table 1 ▸. Coordinates and structure factors have been deposited in the Protein Data Bank with accession codes 6i56 (XepA form I), 6ia5 (XepA form II) and 6i5o (YomS).

### 2.5. *In vivo* cytotoxicity assays   

XepA, YomS, XlyA and XlyB were dialysed after purification into 20 m*M* HEPES pH 7.4 for *in vivo* activity assays. For plaque assays, 10 µl of the proteins at a concentration of 1 mg ml^−1^ were spotted onto 10 ml LB–agar plates [0.75%(*w*/*v*) agar] that contained 500 µl of concentrated bacterial cells. Eight bacterial strains were tested: *Bacillus megaterium* ATCC 14581, *B. subtilis* subsp. *spizizenii* ATCC 6633, *B. pumilus* KPD 181, *B. thuringiensis* KPD 114, *B. mycoides* KPD 15, *Micrococcus luteus* ATCC 4698, *E. coli* MG1655 and *B. subtilis* 168 DSM 23778. Cells were prepared by growing them to an OD_600 nm_ of 0.3 in 25 ml LB. Bacterial cultures were centrifuged at 5000*g* for 30 min at 4°C and the pellet was washed in 20 m*M* HEPES pH 7.4. The washed pellet was resuspended in 500 µl HEPES buffer added to the 50°C semi-solid LB–agar. The plates were left for 20 min at room temperature to set before spotting the protein solutions and were subsequently incubated overnight at 37°C. Hen egg-white lysozyme (HEWL) was applied as a positive control, whereas the negative control was HEPES buffer (20 m*M*, pH 7.4). A summary of the results of the *in vivo* assays can be found in Table 2 ▸.

## 3. Results   

### 3.1. Sequence analysis   

The putative lysins XepA and YomS align very well, with a sequence identity of 38% (54% similarity) covering 85% of the overall sequence (Supplementary Fig. S1). However, neither XepA nor YomS shows significant sequence similarity to the lysins XlyA and XlyB, indicating that these two enzymes have different functionalities and/or mechanisms. In database searches no assigned enzymatic domains could be found for the XepA and YomS sequences. However, YomS corresponds to the C-terminal half of XepA. A sequence search for the N-terminal XepA domain in the SPβ prophage genome in the *B. subtilis* database SubtiWiki (Michna *et al.*, 2016 ▸) did not identify a protein with similar sequence. Both proteins, XepA and YomS, also align well with the *B. subtilis* 168 skin element prophage protein YqxG, as found in SubtiWiki and annotated with unknown function. For XepA the sequence identity is 56% (100% cover, 69% similarity), whereas the identity for YomS is 33% (83% cover, 56% similarity). The high sequence identity to *B. subtilis* YqxG suggests that this protein might have a very similar three-dimensional assembly to the XepA structure described below. The XepA and YomS protein sequences were also screened for transmembrane regions but none were identified, thus indicating that both proteins are soluble components of the lytic system.

### 3.2. Stability and ligand-binding assays   

In addition to XepA and YomS, the lytic enzymes XlyA and XlyB were subjected to thermal shift assays (TSAs). XepA, XlyA and XlyB are significantly more thermostable, with *T*_m_ values of 63, 68 and 62°C, respectively, compared with only 45°C for YomS. XepA and YomS showed comparable thermal stability over a wide pH range, and in both cases a high salt concentration increases the thermal stability slightly. Hence, a range of commercially available screens were employed for crystallization. Additional TSAs were performed to determine potential binding partners of the proteins. Hen egg-white lysozyme (HEWL), which as an *N*-acetylmuramide glycanhydrolase attacks the peptidoglycan at the glucose (Vocadlo *et al.*, 2001 ▸), was used as a positive control. It is known that HEWL binds to triacetylchitotriose (GlcNAc)_3_, and TSAs with 0.05 m*M* HEWL and (GlcNAc)_3_ (16 m*M*) show an increase in the melting temperature, *T*_m_, from 68 to 77°C. The same experiments with d-GlcNAc (*N*-acetyl-d-glucosamine) did not show a stabilization effect. All four proteins were tested for (GlcNAc)_3_ and d-GlcNAc affinity. A similar stabilization (Δ*T*_m_ = 2°C) was observed for XlyA and XlyB in TSAs with (GlcNAc)_3_. For XepA and YomS, however, no stabilizing effect was observed in the presence of these compounds, which suggests that any XepA/YomS lytic action is likely to be based on a different mechanism to the HEWL muramidase activity.

### 3.3. Crystal structure of XepA   

**Figure 2:**
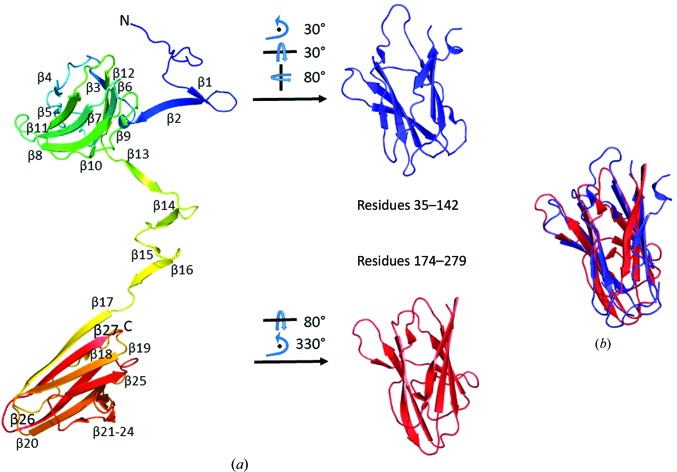
Ribbon diagram of the XepA crystal structure. (*a*) The monomeric unit, which is shown in rainbow colours from blue (N-terminus) to red (C-terminus) with annotation of all strands, reveals two β-sandwich folds that are connected by a linker region. The truncated N-terminal domain (blue) and C-terminal domains (red) are depicted in a reoriented position with the C^α^ atoms used for least-squares superpositioning. (*b*) The β-­sandwiches can be superimposed with an r.m.s.d. of 2.5 Å.

**Figure 3:**
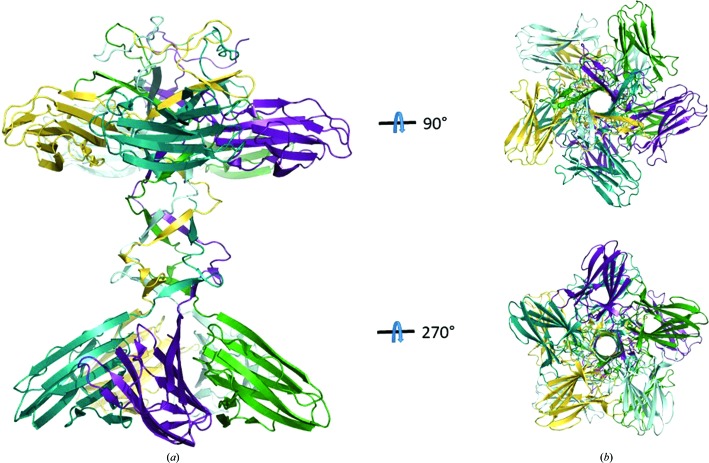
Ribbon diagram of the crystal structure of the XepA pentamer (*a*) with each polypeptide chain depicted in a different colour shows a dumbbell-shaped structure in which two discs are connected by a linker region. (*b*) Top view of the N-terminal domain and bottom view of the C-terminal domain of the XepA pentamer.

**Figure 4:**
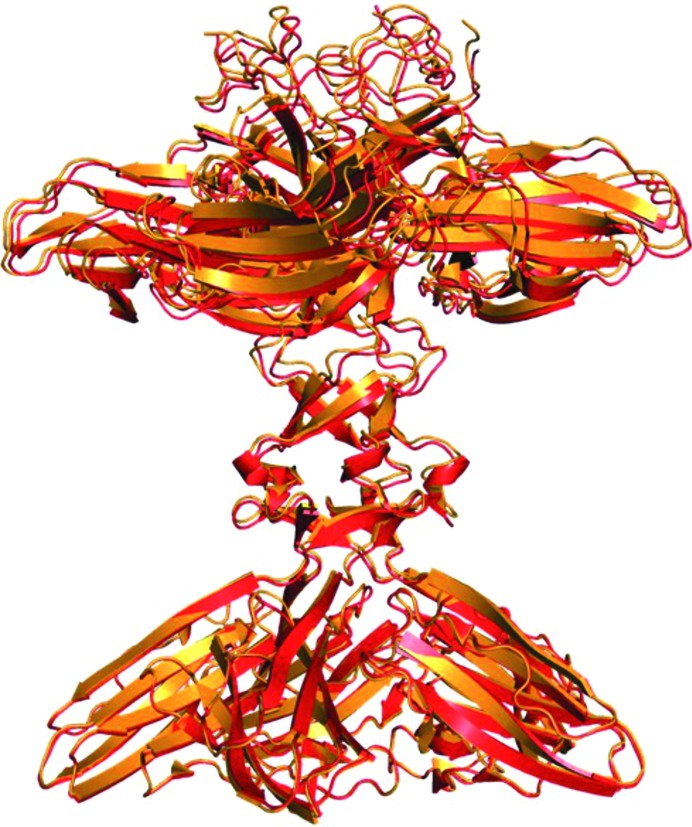
Least-squares superposition of the XepA pentamer in two crystal forms (form I in red and form II in orange). Only the C-terminal pentamer was used to calculate the transformation, which was then applied to the full pentamer. This operation reveals a domain shift that corresponds to a rotation of 2.1° of the N-terminal discs with respect to one another.

**Figure 5:**
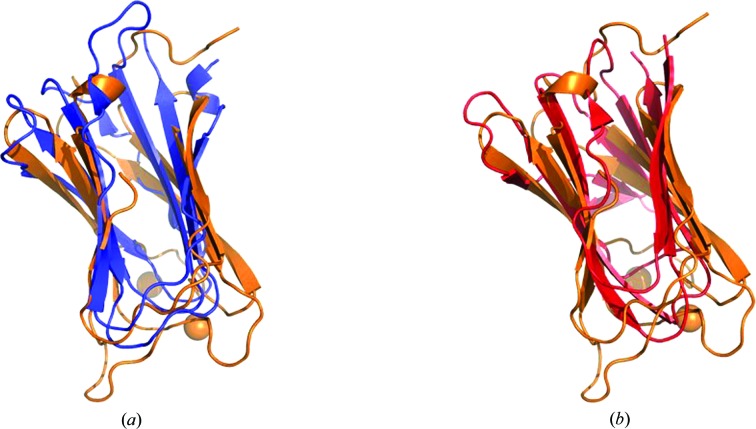
Least-squares superpositions of the β-sandwich folds of XepA with the C2 domain of the α-toxin from *C. perfringens* (PDB entry 2wxt) in orange: (*a*) the N-terminal XepA domain in blue (r.m.s.d. on C^α^ atoms of 2.9 Å), (*b*) the C-terminal XepA domain in red (r.m.s.d. on C^α^ atoms of 3.0 Å).

XepA crystallized from several conditions in multiple crystal forms. The crystal structures of the two highest diffracting crystal forms are reported here (Table 1 ▸). In both crystal forms XepA forms two domains of antiparallel 4 + 4 β-sandwich folds (jelly rolls) which are linked by a 30-amino-acid connector (Fig. 2 ▸ *a*). Superpositions of the N-terminal (residues 35–142) and the C-terminal (residues 174–279) β-sandwiches show that they are similar in fold but have major differences (r.m.s.d. of 2.5 Å; Fig. 2 ▸ *b*). In the crystal, XepA forms a highly symmetric pentamer (Fig. 3 ▸) with a dumbbell-shaped structure. The N- and C-terminal domain pentamers are discs connected by a tunnel-like linker region, which is about 10 Å wide and 45 Å long. The total length of the molecule is roughly 100 Å. The subunits in the pentameric structure adopt very similar structures, with r.m.s.d.s ranging from 0.4 to 0.9 Å when superimposed. The interactions of the β-sandwich moieties within the N- and C-terminal discs are distinctly different and result in a more planar (N-terminal) and a conical (C-terminal) disc. Comparing the XepA structures in different crystal forms, only minor differences in the overall fold can be observed. A superposition of the whole pentamer of XepA crystal forms I and II resulted in an r.m.s.d. of 0.9 Å. However, using only the C-terminal pentamer disc of the different crystal forms to calculate the superposition matrix (r.m.s.d. of 0.3 Å), the molecule shows a domain motion in the tunnel and N-terminal disc corresponding to a rotation of 2.1° (Fig. 4 ▸). This indicates considerable flexibility between the two discs, which may be important for the protein function. Since the individual chains in each crystal form are very similar, the highest resolution structure is used in the following discussions. The β-sandwich fold in XepA is very similar to a C2-domain fold, which is seen in proteins that interact with the cytoplasmic membrane. A superposition with the C2 domain of *Clostridium perfringens* α-toxin (PDB entry 2wxt; Naylor *et al.*, 1999 ▸; Vachieri *et al.*, 2010 ▸) is presented in Fig. 5 ▸. The r.m.s.d.s of these superpositions are 2.9 Å for the N-terminal domain and 3.0 Å for the C-terminal domain. However, unlike in the α-toxin C2 domain, no Ca^2+^ ions were found in XepA.

### 3.4. Crystal structure of YomS   

**Figure 6:**
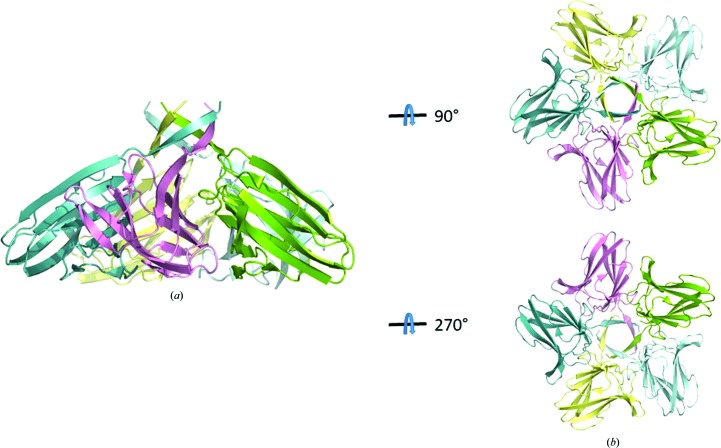
Ribbon diagram of the crystal structure of the YomS homopentamer. (*a*) Each polypeptide chain is depicted in a different colour. (*b*) Top view of the YomS pentamer and bottom view of the YomS pentamer.

**Figure 7:**
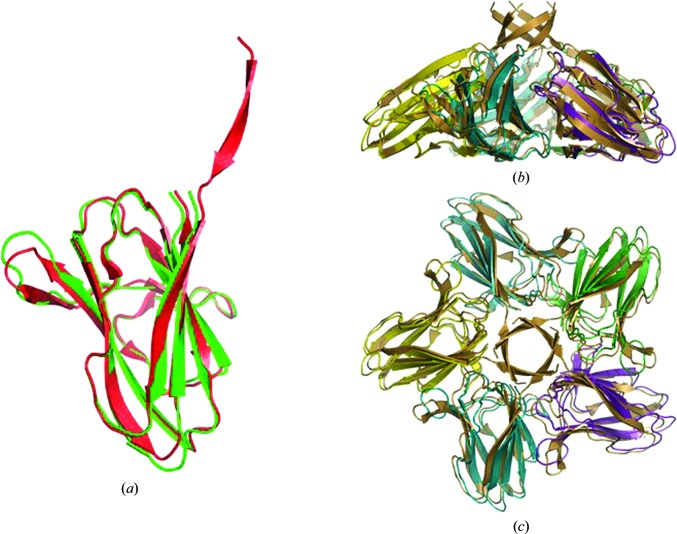
Ribbon diagrams of least-squares superpositions of (*a*) one YomS monomer (red) on the C-terminal domain of an XepA monomer (green; r.m.s.d. of 0.6 Å) and (*b*) the whole YomS pentamer (brown) on the XepA C-terminal pentameric disc (chains depicted in different colours; r.m.s.d. of 1.2 Å).

The three-dimensional structure of YomS was determined to a resolution of 1.3 Å by SeMet SAD phasing. YomS adopts an antiparallel β-sandwich fold, which arranges as a pentameric disc similar to the C-terminal domain of XepA (Fig. 6 ▸). In this disc the monomers can be superimposed on each other with an r.m.s.d. of 0.1–0.2 Å. Comparing YomS with the C-terminal domain of XepA shows that the structures are highly conserved, as presented in Fig. 7 ▸. The monomer sub­units superimpose with an r.m.s.d. of 0.5–0.6 Å on the XepA C-terminal disc moieties (Fig. 7 ▸ *a*), whereas the full pentamers superimpose with an r.m.s.d. of 1.2 Å (Fig. 7 ▸ *b*). Although the sequence identity between XepA and YomS is only 38%, the three-dimensional structures of YomS and the C-terminal XepA domain are highly conserved, supporting the notion that the domains in each protein serve similar functionalities.

### 3.5. Pentamer interfaces   

The individual interfaces within the pentamers of both proteins show extensive electrostatic interactions. The buried surfaces between two XepA chains and two YomS chains amount to approximately 3300 and 1100 Å^2^, respectively. Rather than being based on one or two key residues, the interfaces are mainly held together by a large number of hydrogen bonds (over 40 for XepA and approximately 20 for YomS) and salt bridges.

### 3.6. Cytotoxicity   

**Figure 8:**
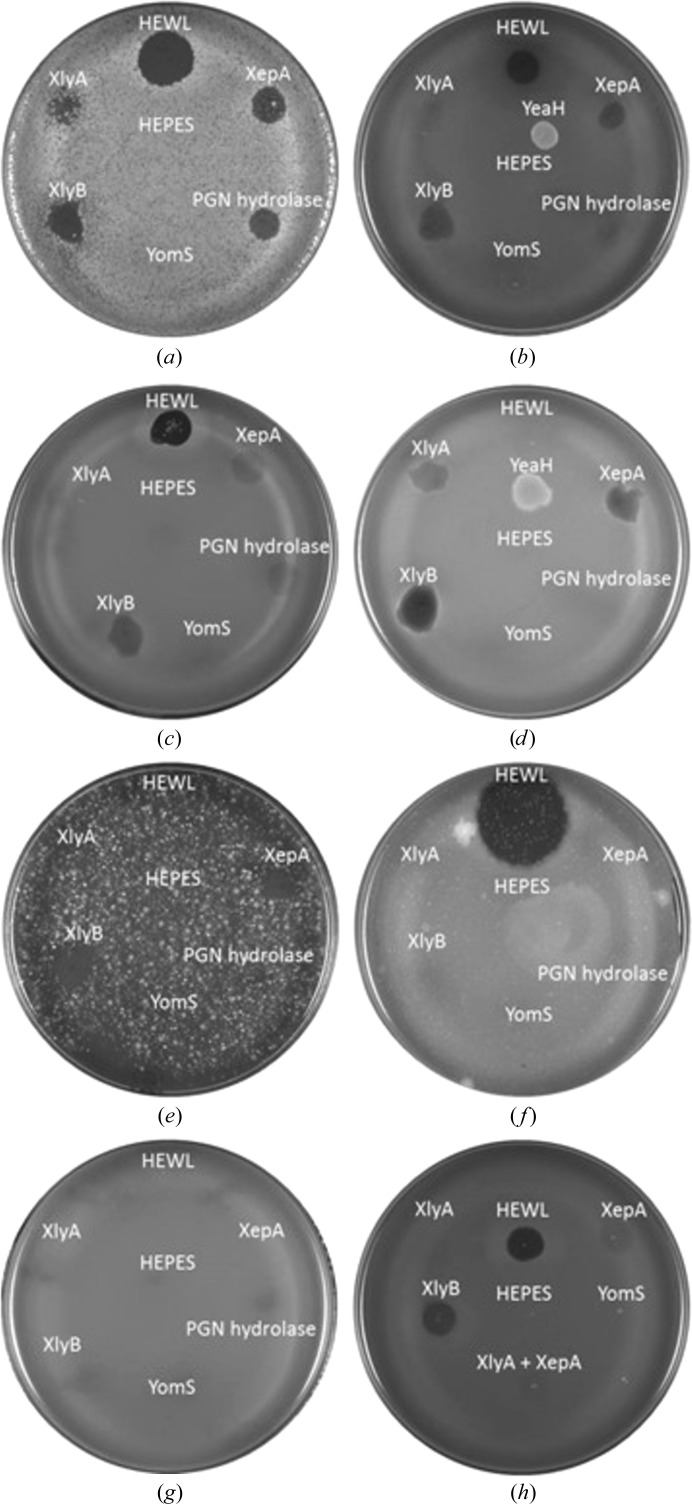
Cytotoxicity of target enzymes. Bacterial plate assays of XepA, YomS, XlyA and XlyB. HEWL and HEPES buffer (20 m*M*, pH 7.4) were used as positive and negative controls, respectively. (*a*) *B. megaterium* ATCC 14581, (*b*) *B. subtilis* subsp. *spizizenii* ATCC 6633, (*c*) *B. pumilus* KPD 181, (*d*) *B. thuringiensis* KPD 114, (*e*) *B. mycoides* KPD 15, (*f*) *M. luteus* ATCC 4698, (*g*) *E. coli* MG 1655, (*h*) *B. subtilis* 168 DSM 23778. The activities of the following proteins were also tested: the YeaH protein of unknown function from *B. subtilis* and PGN hydrolase, a putative lytic enzyme from *B. subtilis* phage vB_BsuP-Goe1.

As the target proteins had been described to play a role in the lytic systems of their prophages, they were tested for cytotoxic activity in bacterial plate assays (Fig. 8 ▸). Purified proteins were applied onto mid-log phase bacterial culture plates and incubated overnight at 37°C. HEWL was used as a positive control and HEPES buffer as a negative control. Eight different bacterial cultures (*B. megaterium*, *B. pumilus*, *B. subtilis* subsp. *spizizenii*, *B. thuringiensis*, *B. mycoides*, *M. luteus*, *E. coli* and *B. subtilis* 168) were tested and the results are summarized in Table 2 ▸. The positive control HEWL shows lytic activity with the Gram-positive bacteria *B. megaterium*, *B. pumilus*, *B. subtilis* subsp. *spizizenii*, *M. luteus* and *B. subtilis* 168, whereas XepA and XlyB show plaque formation with the same samples and in addition with *B. thuringiensis* and *B. mycoides*. The *N*-acetylmuramoyl-l-alanine amidase XlyA only shows lysis with *B. megaterium* and *B. thuringiensis*. YomS does not exhibit any effects on the tested bacteria. As the crystal structures revealed a high degree of structural similarity of the C-terminal XepA domain and YomS, truncated mutants of XepA were designed and the variants were produced. They included (i) only the N-terminal protein (XepA_N), (ii) the N-terminal domain including the linker region (XepA_NL) and (iii) only the C-terminal domain (XepA_C). *In vivo* plate assays with these variants and *B. megaterium* (Supplementary Fig. S2) displayed no activity for XepA_N and XepA_C. XepA_NL shows minimal activity. This clearly indicates that the full-length XepA protein is required for cytotoxic activity.

## 4. Discussion   

### 4.1. Ligand binding and biological activity   

All prophages rely on a functional lytic system to release their progeny into the host. The enzymes of the lytic system need to target both the cytoplasmic membrane and the peptidoglycan. In our studies, we exposed mainly Gram-positive bacterial cultures, namely different *Bacillus* species and *M. luteus*, and the Gram-negative *E. coli* to four proteins with putative lytic activity. As a positive control, the known lytic enzyme HEWL was tested as an *N*-acetylmuramide glycanhydrolase. The Gram-negative *E. coli* did not show any disruption of the bacterial cells, which means that none of the proteins were able to attack the bacterial outer membrane. Interestingly, XepA showed lytic activity against all *Bacillus* species tested (Table 1 ▸), very similar to the results for XlyB, which was previously identified as an *N*-acetylmuramoyl-l-alanine amidase. HEWL as a positive control was not active against *B. thuringiensis* and *B. mycoides*, but was the only enzyme that shows lytic activity towards *M. luteus*. XlyA only displays lytic activity towards two bacterial strains: *B. megaterium* and *B. thuringiensis*. In contrast, the SPβ prophage YomS protein alone does not display detectable cytotoxic activity against any of the selected bacteria. To fully understand which bacteria are targeted by which lysins, detailed knowledge of the cytoplasmic membrane, the peptidoglycan layer and eventually of the bacterial capsule is required, as these characteristics are species-specific and very diverse. This represents one key to the enzymatic selectivity in lytic systems towards bacteria. Although XlyA and XlyB differ in their lytic activity towards the selected *Bacillus* strains, both proteins are known *N*-acetylmuramoyl-l-alanine amidases with a typical lysin composition (Supplementary Fig. S3): a C-terminal cell-wall-binding domain (CBD) and an N-terminal catalytic domain with a Zn^2+^-dependent active site (Low *et al.*, 2011 ▸). In the thermal shift assays we established that XlyA and XlyB, and also HEWL, are stabilized by (GlcNAc)_3_ binding, which corresponds to their ability to attack the peptidoglycan layer during the lytic process. XepA and YomS, on the other hand, do not bind the same substrate and hence it is likely that they would employ a different mechanism for any lytic activity. In addition, the crystal structure of the catalytic domain of XlyA (PDB entries 3rdr and 3hmb; Low *et al.*, 2011 ▸) shows no structural similarity to either XepA or YomS. In summary, we show that XepA initiates cell death *in vivo*, whereas YomS has no effect. We also showed that it is unlikely that XepA attacks the peptidoglycan in the same manner as do the amidases XlyA, XlyB and HEWL.

### 4.2. From structure to function   

XepA forms a remarkable pentameric structure in which two disc-shaped domains are connected by a linker region. As shown above, the C-terminal disc of XepA is very similar to the structure of YomS, and the full-length protein including both domains is essential for cytotoxic activity. Both the XepA and YomS monomeric β-sandwiches adopt a fold resembling the C2 domain, which usually contains about 110 amino acids and 2–3 Ca^2+^ ions. C2 domains are typically phospholipid-binding and are often involved in cytoplasmic membrane trafficking. Tandem C2 domains are observed in membrane-trafficking proteins, for example synaptotagmins (Xu *et al.*, 2014 ▸). This structural similarity could explain the function of the XepA and YomS β-sandwich structures. Although C2 domains are mostly observed in eukaryotic proteins coupled to enzymatic domains (Cho, 2001 ▸), there are a few prokaryotic examples of C2 domains. The *C. perfringens* and * C. absonum* α-toxins consist of C2 domains bound to phospholipase domains (Clark *et al.*, 2003 ▸). The C2 domains in these examples bind Ca^2+^ ions depending on the cytoplasmic domain, but not all C2 domains are Ca^2+^-dependent. In the XepA and YomS crystal structures described here no Ca^2+^ ions were identified. A Ca^2+^-independent interaction has been described to rely on a lysine-rich cluster of Rabphilin 3A with the cytoplasmic membrane (Guillén *et al.*, 2013 ▸). Multiple lysine residues at the XepA N-terminus (Supplementary Fig. S1) could be involved in similar interactions. As XepA displays two domains with very similar folds, a tandem C2 domain is also possible.

Looking at the three-dimensional structure of XepA with the linker region connecting the N-terminal and C-terminal pentameric discs, it appears possible that the structure may form a tunnel involved in DNA release. However, the absence of a transmembrane domain and the relatively small size of the formed pore discourage this notion. Comparisons with viral portal proteins (Sun *et al.*, 2015 ▸; McElwee *et al.*, 2018 ▸) show that the XepA tunnel has a relatively small diameter of approximately 10 Å, which suggests that it is too small to allow the entry of any DNA molecule (dsDNA, 20 Å; ssDNA, 15 Å). Although it may be conceivable that high flexibility in the XepA linker region may permit a conformational change, for example a rotational movement of the C-terminal and N-terminal discs to open the pore area, this would require a major structural realignment.

The β-sandwich or β-jelly roll is also a common viral capsid-protein motif in non-enveloped viruses (Bamford *et al.*, 2005 ▸). In bacteriophage PRD1, the P31 penton protein is described to have a jelly-roll topology. A pentamer of P31 occupies the vertices in the icosahedral viral capsid shell (Abrescia *et al.*, 2004 ▸) and builds the base of the vertex spikes in PRD1 on pentagon faces. Through their P31 inter­action, two other proteins (P5 and P2) allow spike formation (Sokolova *et al.*, 2001 ▸). The P31 jelly rolls are in vertical alignment in these pentamers. A 4 Å resolution X-ray crystal structure of the PRD1 bacteriophage has been deposited in the PDB as entry 1w8x, in which chain *N* corresponds to P31. Similar penton proteins have been reported in other untailed viruses, but not in Caudovirales, where in most cases the major capsid protein forms hexameric and pentameric capsomers which are arranged on the capsid surface (Fokine & Rossmann, 2014 ▸). Although the sequence similarity to other penton proteins is low, the pentameric assembly of XepA and YomS points towards a similar location in the *B. subtilis* prophage capsids. However, the orientations of the XepA and YomS jelly rolls in the structures reported here are different, with an approximate angle of 45° to a binding surface. The absence of XepA in the mature phage would be contraindicative to the assumption that it is part of the viral capsid. However, the XepA C-terminal domain and YomS may bind to the capsid in a vertex position according to their fivefold symmetry, stabilizing the virus particle (YomS) and helping the virus particle to escape (XepA). Many phages have additional domains lying on the capsid surface, which can be seen in cryo-EM reconstructions as protrusions. In the tailed bacteriophage φ29, for example, head fibres decorate the phage head (Xiang & Rossmann, 2011 ▸). The fibre bases of φ29 are described as trimers of two small β-barrel subdomains (Xu *et al.*, 2019 ▸). In the tailed bacteriophage T4, the outer capsid proteins Hoc and Soc decorate the capsid surface (Rao & Black, 2010 ▸). As XepA was observed during phage maturation and not in the mature phage, one of its roles might be capsid reinforcement during capsid formation. Cement proteins that reinforce major capsid-protein interactions have been reported to have a similar jelly-roll topology, as shown in the structure of *Bordetella* bacteriophage BPP-1 (Zhang *et al.*, 2013 ▸).

### 4.3. Mechanism of cell lysis   

**Figure 9:**
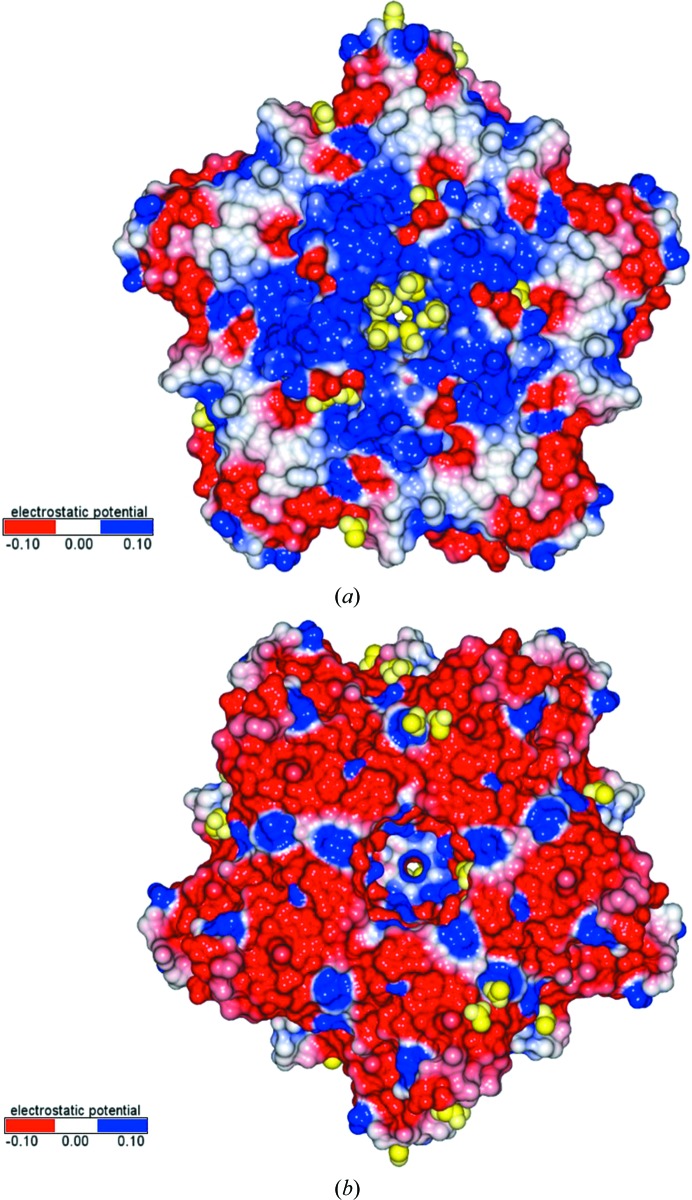
Electrostatic surfaces of the XepA pentamer calculated using *CCP*4*mg* (McNicholas *et al.*, 2011 ▸): red, −0.10 V e^−1^; white, 0.00 V e^−1^; blue, 0.10 V e^−1^. (*a*) View of the N-terminal disc, which is mostly positively charged. Acetate ions and glycerol molecules depicted in CPK representation (yellow) bind predominantly in the tube region. (*b*) View of the C-terminal disc, which shows a prevalently negatively charged surface.

DNA phages can adopt multiple strategies to accomplish host-cell lysis, including the holin-dependent export of lysins, the Sec-mediated export of lysins with signal peptides (as observed in fOg44) and the holin-independent export of lysins with SAR (signal–arrest–release) as observed in coliphage P1 (Catalão *et al.*, 2013 ▸; Fernandes & São-José, 2018 ▸). XepA contains structural domains that are typically associated with cytoplasmic membrane binding. The generally positively charged surface at the XepA N-terminus (Fig. 9 ▸ *a*) may be able to bind to the negatively charged phosphate moieties of the phospholipid bilayer. Furthermore, the crystal structure of XepA reveals acetate ions from the crystallization solutions that bind on the electropositive surface in the centre of the dumbbell-shaped protein, showing potential phosphate-binding sites. In contrast, the XepA C-terminal surface is almost exclusively electronegatively charged (Fig. 9 ▸ *b*), which suggests that the same interaction is not feasible between the C-terminal surface and the cytoplasmic membrane.

Like all members of the Caudovirales, PBSX and SPβ form an icosahedral head made of hexamers and pentamers of its major coat protein (MCP). Considering the conserved fivefold symmetry in the protein structures described here, it is feasible that the C-terminal pentamer of XepA as well as the structurally very similar YomS may bind at least transiently to the prophage capsid proteins. The XepA pentamer might dock onto the phospholipid membrane with its N-terminal positively charged surface and hence disrupt the proton motive force (PMF) in a similar way to pinholins (Catalão *et al.*, 2013 ▸). Pinholins cause small lesions in the cytoplasmic membrane and in this way activate the host-cell lytic system (Pang *et al.*, 2009 ▸). A major difference to the pinholins is that XepA does not possess a transmembrane domain, since the surface of the linker region is clearly hydrophilic (Supplementary Fig. S4) and thus cannot be integrated into the cytoplasmic membrane.

We hypothesize that XepA may be located on the phage capsid, binding to the cytoplasmic membrane and dissipating the proton motive force of the membrane. XepA could thereby interfere with the host-cell secretion machinery and/or subvert the bacterial lytic system to induce host-cell lysis, as shown previously (Fernandes & São-José, 2018 ▸). Ultimately, XepA supports cell lysis and allows the release of assembled virus particles in the phage lytic cycle.

## 5. Related literature   

The following references are cited in the supporting information for this article: Larkin *et al.* (2007 ▸) and Robert & Gouet (2014 ▸).

## Supplementary Material

PDB reference: YomS, 6i50

PDB reference: XepA, 6i56

PDB reference: 6ia5

Supplementary Tables and Figures. DOI: 10.1107/S2059798319013330/gm5068sup1.pdf

## Figures and Tables

**Table 1:** Data-collection and refinement statistics Values in parentheses are for the highest resolution shell.

	XepA (form I)	XepA (Tb derivative)	XepA (form II)	YomS	SeMet-YomS
Data collection					
Beamline	I03, DLS	I04, DLS	I24, DLS	I04, DLS	P13, EMBL/DESY
Space group	*P*2_1_2_1_2_1_	*P*2_1_2_1_2_1_	*P*2_1_2_1_2_1_	*C*2	*C*2
Unit-cell parameters					
*a* (Å)	85.81	91.34	90.61	107.00	107.83
*b* (Å)	106.47	126.62	126.03	52.16	49.57
*c* (Å)	158.84	152.02	151.46	106.74	100.88
β (°)	90	90	90	95.97	92.31
Wavelength (Å)	0.9795	1.649	0.9772	0.9795	0.9795
Resolution (Å)	29.8–2.12 (2.16–2.12)	30–2.50 (2.56–2.50)	96.9–1.88 (2.07–1.88)	53.08–1.33 (1.36–1.33)	48.33–2.00 (2.05–2.00)
No. of observations	834395 (46330)	1040740 (41768)	1118622 (33451)	662174 (49107)	490568 (33696)
*R*_merge_	0.148 (2.07)	0.156 (2.60)†	0.080 (0.79)	0.088 (1.40)	0.098 (0.341)†
*R*_p.i.m._	0.049 (0.676)	0.053 (1.30)†	0.024 (0.319)	0.044 (0.693)	0.040 (0.141)†
〈*I*/σ(*I*)〉	11.0 (1.2)	14.1 (0.8)	17.2 (1.8)	9.8 (1.1)	19.1 (7.4)
CC_1/2_	0.998 (0.513)	0.999 (0.354)	0.998 (0.683)	0.998 (0.498)	0.998 (0.981)
Completeness	1.000 (1.000)	0.999 (0.997)‡	0.771 (0.213)/0.922 (0.616)§	0.999 (1.000)	0.999 (0.985)†
Multiplicity	10.0 (10.2)	16.9 (9.3)‡	11.2 (6.7)	4.9 (5.0)	13.5 (12.8)
No. of heavy atoms		5 Tb			5 Se
Refinement					
*R*_work_/*R*_free_	0.173/0.221		0.172/0.212	0.154/0.178	
No. of atoms	10657		10842	4310	
Ligands	6 glycerols		11 glycerols, 22 acetates	None	
No. of waters	861		1026	851	
R.m.s.d., bonds (Å)	0.013		0.007	0.016	
R.m.s.d., angles (°)	1.68		1.43	1.90	
Ramachandran plot					
Favoured (%)	98.1		97.1	98.7	
Allowed (%)	100		99.2	99.2	

† *R*_merge_ within (*I* ^+^/*I* ^−^).

‡ Anomalous completeness.

§ Ellipsoidal/spherical completeness.

**Table 2:** Summary of the cytotoxic activity of XepA, YomS, XlyA and XlyB on a selected range of bacterial cultures Plaque zone: +++, >7 mm; ++, 4–7 mm; +, 1–4 mm; −, none.

Species	HEWL	Control	XepA	YomS	XlyA	XlyB
*B. megaterium* ATCC 14581†	+++	−	++	−	+	++
*B. subtilis* subsp. *spizizenii* ATCC 6633†	++	−	+	−	−	++
*B. pumilus* KPD 181†	+++	−	+	−	−	++
*B. thuringiensis* KPD114†	−	−	++	−	++	+++
*B. mycoides* KPD 15†	−	−	++	−	−	++
*M. luteus* ATCC 4698†	+++	−	−	−	−	−
*E. coli* MG1655‡	−	−	−	−	−	−
*B. subtilis* 168 DSM 23778†	+++	−	+	−	−	++

† Gram-positive species.

‡ Gram-negative species.
