# Supplementary material for: Crystal structures of the Bacillus subtilis prophage lytic cassette proteins XepA and YomS
Source: Acta Crystallogr D Struct Biol. 2019 Nov 1;75(Pt 11):1028–39. doi: 10.1107/S2059798319013330 (PMC6834076; doi:10.1107/S2059798319013330)
Supplement: Supplementary file 1 [file d-75-01028-sup1.pdf]

STRUCTURAL
BIOLOGY

Volume 75 (2019)

Supporting information for article:

Crystal structures of the *Bacillus subtilis* prophage lytic cassette proteins XepA and YomS

Stefanie Freitag-Pohl, Andrius Jasilionis, Maria Håkansson, L. Anders Svensson, Rebeka Kovačič, Martin Welin, Hildegard Watzlawick, Lei Wang, Josef Altenbuchner, Magdalena Płotka, Anna Karina Kaczorowska, Tadeusz Kaczorowski, Eva Nordberg Karlsson, Salam Al-Karadaghi, Björn Walse, Arnthór Aevarsson and Ehmke Pohl

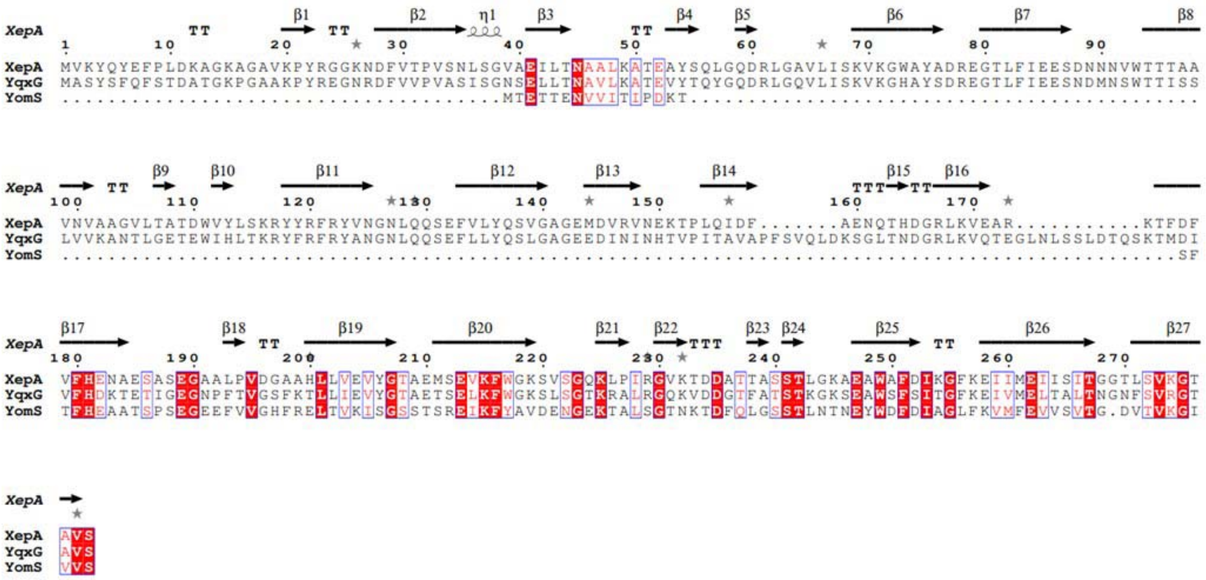

Figure S1 Sequence alignment of the putative lysins XepA, YomS and YqxG. The secondary structure annotation in the top line is based on the crystal structure of XepA presented here.

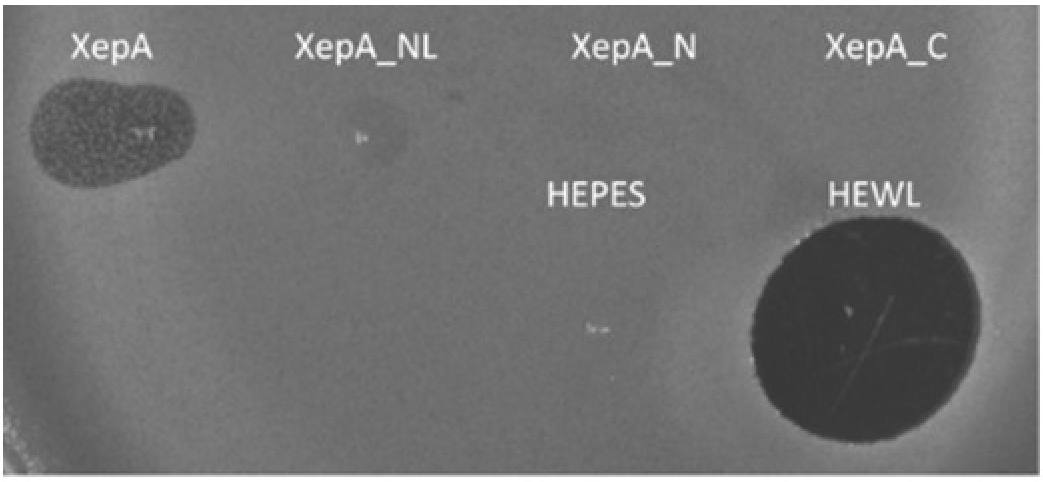

Figure S2 Cytotoxicity of XepA truncated variants. *In vivo* assays of XepA truncated variants XepA_NL, XepA_N and XepA_C on *Bacillus megaterium* ATCC 14581. Wild-type XepA and HEWL served as positive controls, HEPES buffer (20 mM, pH 7.4) was used as a negative control. Out of three truncated XepA variants tested, only XepA_NL shows minimal activity.

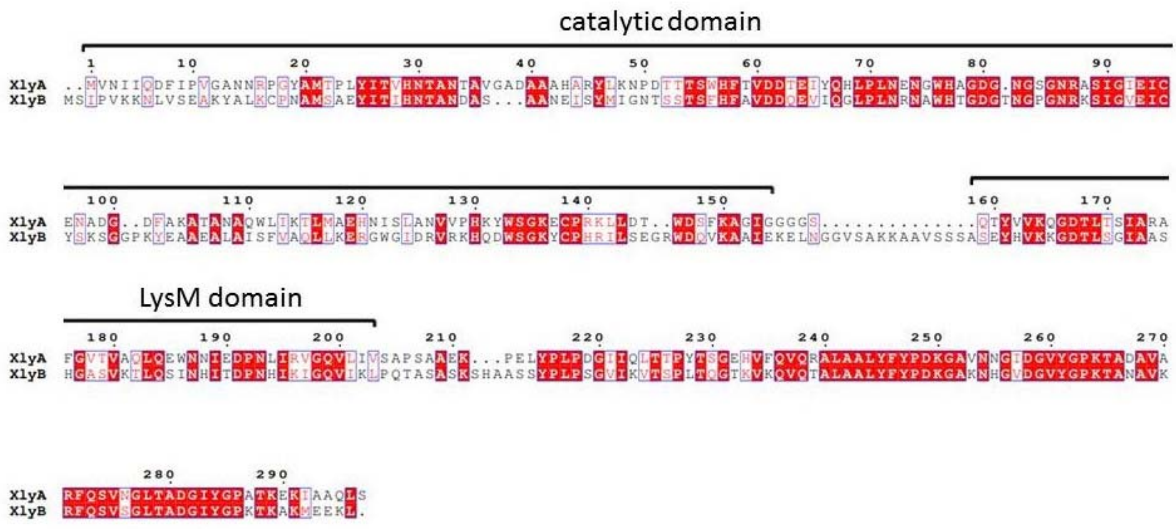

Figure S3 Sequence alignment of the lysins XlyA and XlyB with ClustalW (Larkin et al. 2007) and ESPript (Robert & Gouet, 2014). The residues corresponding to the cell wall binding LysM domain are 159 to 203 (XlyA) and 177 to 222 (XlyB).

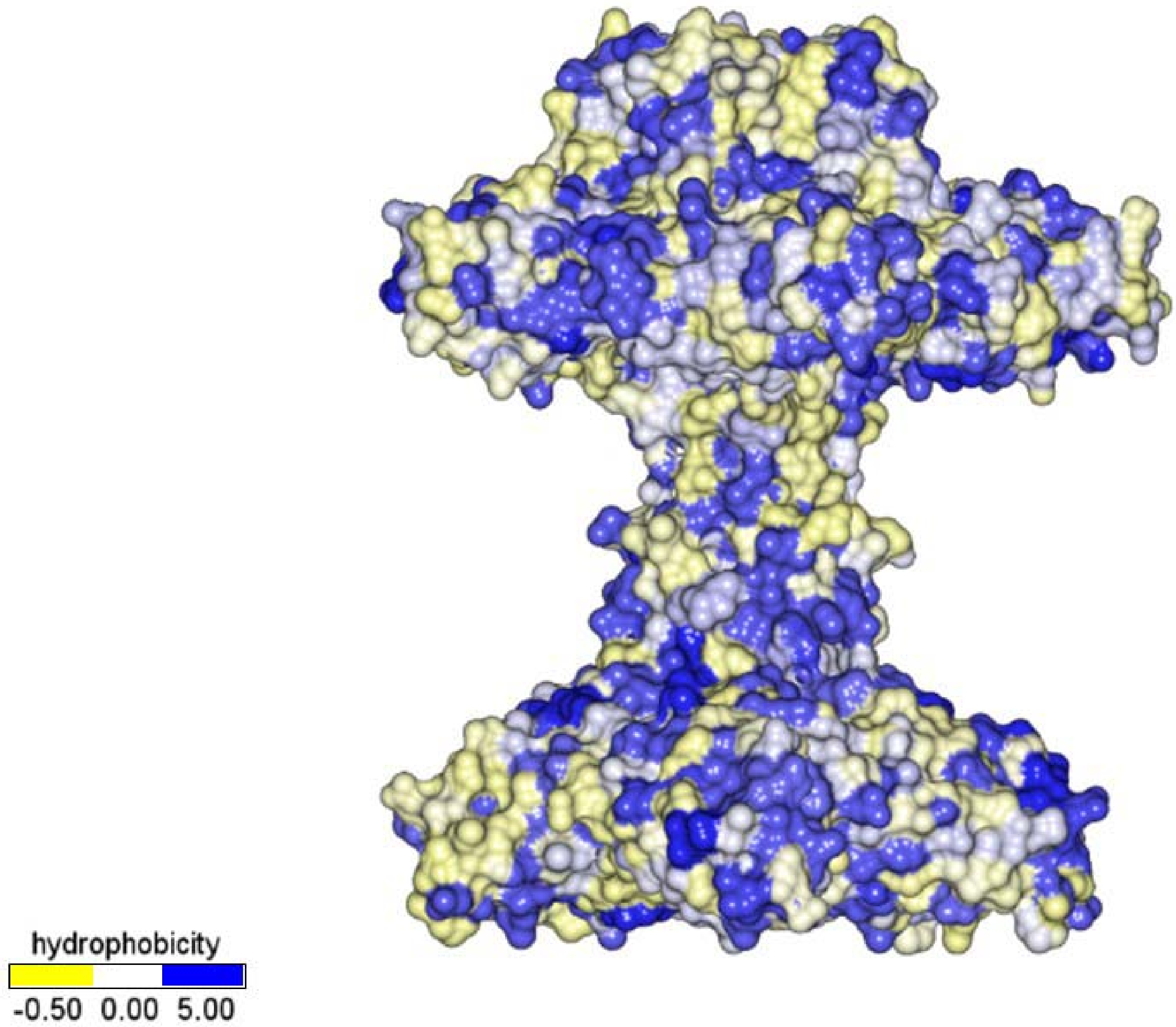

Figure S4 Surface representation of hydrophobicity of the XepA pentamer for its side view (CCP4mg) shows that the linker region is not entirely hydrophobic and thus does not resemble a trans-membrane region.
